# Two More Anæsthetic Fatalities

**Published:** 1907-07-06

**Authors:** 


### TWO MORE ANÆSTHETIC FATALITIES.

Only four weeks ago we considered at some length in these columns some remarks of Dr. Waldo, Coroner for the City and for Southwark, on the question of fatalities during anæsthesia. Since then that gentleman has unfortunately been required to deal with two more such cases, and it is with very considerable amazement that we observe an article in one of the more sensational of the daily papers suggesting in consequence of one of these inquests, that coroners should not be medical men. It is true that this suggestion is said to emanate from a "well known doctor," but anything more preposterous is difficult to imagine than the doctrine laid down "that they" (*i.e.*, coroners) "may be slightly biased by their medical etiquette," whatever that may mean. It is particularly difficult to see why this extraordinary comment should be appended to an account of an inquest by Dr. Waldo, who has for years distinguished himself by the very thorough way in which every death during anæsthesia is investigated in his court. A printed form of questions touching every detail of each fatality is supplied to the anæsthetist concerned, and when returned to the Coroner is carefully preserved, together with an account of the post-mortem findings, verdict, and other particulars of the case.

In his two latest inquests Dr. Waldo has devoted much attention to certain very important points on which wide difference of opinion seems to prevail, and has elicited some interesting information. The cases occurred in St. Bartholomew's and Guy's Hospitals respectively, and resembled each other in that both were cases of emergency (septic meningitis and hæmorrhage from gastric ulcer respectively), and that both administrators are on their respective regular anæsthetic staffs. At the former institution we find from the evidence that chloroform, ether, and the C.E. mixture are used in that order of frequency, and in the proportions of 25, 15, and 6 : whereas at the latter the order is exactly the reverse, though the precise figures are not quoted.

At St. Bartholomew's chloroform made from ethylic alcohol is alone used, and the anæsthetist from that hospital, who gave evidence, believes that this form of chloroform, which costs in bulk about 5s. per lb., is followed by fewer unpleasant after-effects, and is safer than that made from acetone. Incidentally, he is reported to have expressed a preference for chloroform over the chloroform-ether mixture. On the other hand, Guy's are perfectly satisfied with their acetone chloroform, which costs about 2s. per lb. At the last inquest held the anæsthetist, the surgeon, and the hospital dispenser were unanimous on the point. In this connection may be quoted Dr. Hewitt, who under date January 1907, says :—"It is doubtful whether there is any real advantage in practice in employing chloroform prepared from rectified spirit. The author has used methylated chloroform for many years, and cannot satisfy himself that it is inferior to ethyl-alcohol chloroform. He has also used acetone-made chloroform with equally good results. He is forced to believe that there is little or no substantial foundation for much that has been written concerning the importance of this or that brand." The preponderance of opinion and practice among London hospitals and anæsthetists is, in fact, on the side of Dr. Hewitt and of Guy's, and against St. Bartholomew's. We do not gather from the evidence what kind of ether is used at the latter hospital, but at the former it is that made from methylated alcohol, costing 1s. 10½d. per lb., as against 2s. to 3s. for that from methylated spirit, and 5s. 6d. for that from ethylic alcohol. The difference in the cost of these drugs when prepared from different sources is, of course, no index of the labour required in the processes, but is due to the heavy duty on ethylic alcohol. All the witnesses expressed agreement with the general conclusion of the Report of the Anæsthetics' Committee of the British Medical Association, 1900, which runs :—"From the evidence before the Sub-Commitee they are convinced that by far the most important factor in the safe administration of anæsthetics is the experience which has been acquired by the administrator. In many cases the anæsthetisation completely transcends the operation in gravity and importance, and to ensure success, particularly in these cases, it is absolutely essential that an anæsthetist of large experience should conduct the administration." This embodies a truth so plain that it needs no discussion here; nor does it conflict with any of the points raised in our previous discussion of the same subject. The training and dispersal of as large a number as possible of such "anæsthetists of large experience" is one of the most important functions of the medical schools, and one which they are fulfilling with steadily increasing efficiency. Meanwhile, the thanks of the profession, of the public, and of the medical schools are due to Dr. Waldo for the fair and practical way in which he sets about elucidating to the uttermost every case of these deplorable fatalities.

